# Making Lived-Experience Research Accessible: A Design Thinking Approach to Co-Creating Knowledge Translation Resources Based on Evidence

**DOI:** 10.3390/ijerph18179250

**Published:** 2021-09-02

**Authors:** Katherine M. Boydell, Anne Honey, Helen Glover, Katherine Gill, Barbara Tooth, Francesca Coniglio, Monique Hines, Leonie Dunn, Justin Newton Scanlan

**Affiliations:** 1 Black Dog Institute, Sydney, NSW 2034, Australia; 2 School of Psychiatry, University of New South Wales, Sydney, NSW 2034, Australia; 3 School of Health Sciences, University of Sydney, Sydney, NSW 2006, Australia; anne.honey@sydney.edu.au (A.H.); monique.hines@sydney.edu.au (M.H.); justin.scanlan@sydney.edu.au (J.N.S.); 4 Enlightened Consultants, Redland Bay, QLD 4165, Australia; helen@enlightened.com.au; 5 Consumer-Led Research Network, University of Sydney, Sydney, NSW 2006, Australia; kate@foundationsforsuccess.com.au; 6 The Mental Health Services (TheMHS) Network, Balmain, NSW 2000, Australia; btooth@bigpond.com; 7 Private Practitioner, Sydney, NSW 2000, Australia; franscescad.coniglio@gmail.com; 8 South Eastern Sydney Local Health District, Sydney, NSW 2010, Australia; Leonie.Dunn@health.nsw.gov.au

**Keywords:** lived-experience research, design thinking, knowledge translation, mental health recovery, co-design

## Abstract

Mental health lived-experience research illuminates the perspectives and experiences of people who live with mental illness. However, little is known about how useful people with lived experience of mental illness/distress might find lived-experience research, nor what the best formats are to bring it to their attention. This paper describes the STELLER study (Supporting the Translation into Everyday Life of Lived-Experience Research), which explores the translation of lived-experience research in the lives of people living with mental illness. Our aim was to use a design thinking approach to develop a range of user-friendly formats to disseminate lived-experience research. A staged design thinking approach was used to develop a translation strategy for lived-experience research. We explored empathy via consumer consultation to understand their perspectives on lived-experience research, refined the design aim, research questions and generated ideas with consumers and mental health professionals, identified the evidence based on lived experience-authored journal articles, worked with design students and peer workers to create a suite of resources and developed prototypes tailored to individual settings and clients. Participatory design thinking strategies are essential to identify the best ways to translate evidence-based lived-experience research via accessible, lay-friendly resources targeted to individuals impacted by mental illness. This study is the first to investigate the feasibility and usefulness of bringing the findings of lived-experience research to individuals impacted by mental illness/distress. It provides evidence about a potentially important source of information that can be used to facilitate their recovery.

## 1. Introduction

Lived-experience research in mental health is research that highlights the experiences of people who live with mental health issues and is conducted by researchers with their own lived experience or in research teams that include people with lived experience [1]. This involvement is understood to result in benefits including producing better quality research and enhancing capacity [1,2,3]. The engagement of individuals with lived experience (also referred to as consumers or service users and used interchangeably in this paper) in mental health research is an emerging and growing arena [4,5]. Whilst there has been policy and, in principle, support for such involvement and engagement from governments around the world, lived-experience researchers conducting academic research in collaboration with other consumers and carers remains uncommon [3,6]. There has been a recent rapid growth in the involvement of service users in service transformation initiatives, in mental health research and in the deliberations regarding what constitutes evidence [7]. The value of lived experience for the mental health knowledge base as well as quality improvement of services has been gradually more appreciated, although not without its challenges [8,9,10].

Lived experience-produced research that focuses on identifying the factors relevant to daily life from a lived experience perspective has the potential to assist people to cope with their mental health challenges, however, this lived-experience research is not universally recognised and is often difficult to find and access. Furthermore, most lived experience-generated research is directed at improving understandings of health professionals or policy makers rather than for direct and practical use by mental health consumers in their daily lives [11].

### Beginnings (International Institute for Mental Health Leadership Event)

As part of the International Institute for Mental Health Leadership (IIMHL) conference in 2017 in Sydney, Australia, a series of pre-conference events took place, one of which was a workshop centred on expanding lived experience-led research. The event included about 20 lived-experience scholars in academia as well as other academic scholars. As part of the breakout group process, our small subgroup of six focused on ‘lived-experience expertise’, the collective wisdom of individuals impacted by mental illness. One of our group members, a peer worker, alerted us to the fact that knowledge and experiences of individuals who experience mental illness/distress as articulated in research rarely find their way to those who are not involved in the research world. Some individuals view researchers as intimidating, “higher up” or living in a “different space”. The findings of lived-experience research, however, were recognised as being useful to a wide range of people going about their personal recovery journeys [7,11]. This occurs both through learning from the wisdom, strategies and successes of other consumers and from understanding that others share/have shared similar experiences (not being alone). Our brainstorming session led to the desire to continue to work on a project together post-workshop day—we initially named ourselves the “Enhancing Experiential Literacy” workgroup.

Our workgroup began to meet bimonthly on Saturday mornings. These meetings were conducted virtually, giving us the opportunity to think about ways to get the findings from lived-experience research out to a wide range of people who experience mental illness/distress. Based on our knowledge of evidence-based knowledge translation strategies [12,13] and coupled with our own experiences, we acknowledged that a wide range of methods would be needed, including written lay-summaries, YouTube videos and other creative means. These strategies are available but are infrequently dispersed, often unwieldy, and unlikely to be accessed by many individuals with lived experience who may benefit from the lived experience knowledge contained within. Groups such as those who are hospitalised, homeless, incarcerated, in long-term institutional settings, technologically challenged, or live in rural or remote areas, are particularly unlikely to know about or access lived-experience research.

Getting lived-experience research out to these individuals is challenging. Using Facebook “likes” and other social media is popular with some groups, but inaccessible to others. We recognised the need to use knowledge from marketing, knowledge translation, public health and human-centred design to devise innovative and effective strategies to get lived-experience research into consumers hands—to increase their experiential literacy. This is likely to involve targeting a range of people who have contact with people who access or work within mental health services, such as peer workers, homelessness workers, shelters and drug and alcohol services, presenting information in formats that are easy for them to share and marketing the need to share that information with people who access services on a regular basis.

In order to explore the strategies to get lived-experience research out to individuals who experience mental illness/distress in the best way possible, we began by exploring the extent to which accessible resources currently exist for those with lived experience to access lived-experience research. We examined what the existing research tells us about the best ways to get information out to a wide range of consumers. We conducted informal interviews with individuals impacted by mental illness/distress in our network to help us understand more, by asking them about the best ways of presenting and marketing lived-experience research. Based on this knowledge, we subsequently developed a proposal and received funding for what we called the STELLER (Supporting the Translation into Everyday Life of Lived-Experience Research) Study, our collaboration of lived-experience researchers and other researchers from diverse organisations, including: the University of Sydney, the Black Dog Institute, the Consumer-Led Research Network, the Local Health Network, The MHS Network and Enlightened Consultants. The overall study aims were three-fold: (i) to translate lived-experience research findings into user-friendly attractive resources, (ii) to disseminate those resources to individuals experiencing mental illness/distress via peer workers and (iii) to evaluate their accessibility and usefulness using a non-randomised experimental study. This paper describes the first aim—to translate evidence-based research authored by individuals with lived experience into user-friendly resources. Aims (ii) and (iii) are described elsewhere [14]. Ethical approval was obtained from the Southeast Sydney Local Health District Human Research Ethics Committee.

## 2. Materials and Methods

A staged design thinking approach [15] was used to develop resources based on the evidence base of lived experience on what helps in the day-to-day lives of people experiencing mental health challenges. Design thinking is characterised by five iterative stages: empathise, define, ideate, prototype and test [16,17]. The first phase—empathise—involved consultation with people with lived experience of mental health issues to understand their perspectives on lived-experience research. This consultation involved a snowball approach, drawing on our respective networks and connections. This was followed by phase 2—defining the problem—involving a conference workshop to engage in discussion about how to address the challenge in accessing lived-experience knowledge, refine the design aim, research questions and begin to generate ideas. This was followed by a selection of exemplar lived-experience literature leading to phase 4—prototype—focused on the design and development of resources to share the main messages from the selected research. Phase 5—test—is described elsewhere [14].

## 3. Results

### 3.1. Phase 1: Empathy: Developing an Understanding of End Users

#### Lived Experience and Peer Worker Consultations

The first phase in design thinking is to empathise—to find out about and understand the perspective of the people we were designing for. We had individuals with lived experience on our research team, but they were active in research, so we needed to expand our reach. Consequently, informal interviews were conducted with a group of 14 individuals with lived experience of mental health issues, some of whom were peer workers. The intent was to gain a sense of the extent to which they were aware of and accessed the lived-experience literature, clearly specifying that this is actual research rather than simply one person’s story or narrative. 

Respondents indicated that they drew on a wide range of sources for information to aide their recovery, including mental health professionals, peers, support/advocacy groups, specific mental health programs, various forms of literature, both hard copies and on the Internet, through to drawing on information gained from past experience. They highly valued hearing about the lived experiences of others and thought that lived-experience research could be helpful. They felt that it could help people feel less alone, normalise their experiences and allow them to learn from the experiences of others. Some said that it would help people reframe their experiences or think in different ways, especially non-medical ways. Others talked about finding hope and inspiration, clarifying their own experiences and understanding others who have different experiences. 

Very few searched for experiential literature, and those who came across it did so by accident. Respondents revealed that they felt such experiential literature is important and valuable and validates experience, helps with self-advocacy hope and wellbeing and creates alternate and new knowledge. The interviews highlighted that people who experience mental illness have little opportunity to learn and integrate knowledge that is emerging from lived-experience research. The mental health lived-experience research base contributes a wealth of collective experiential wisdom to the mental health knowledge base. Like much of the academic literature, this knowledge is not easily accessible or known to people who are negotiating the day-to-day impacts of mental distress [14]. Knowledge translation of existing research that is driven or determined by lived experience can play a significant role in assisting people in navigating both their recovery and the systems of care that they may be accessing [18].

### 3.2. Phase 2: Defining the Problem: Stakeholder Roundtable 

Phase 2 in the design thinking process consists of defining the problem or design aim. We identified the problem and these aims based on our collective experience and the feedback we received in phase 1. Our goal was to identify how we might render lived-experience research brilliantly visible, to effectively bring it to the person, make it effortless to find and translate it into user-friendly, attractive and exciting formats. In order to do this, the problem was discussed with clinicians, researchers and individuals with lived experience. This discussion was in the form of a workshop held at a conference focused on mental health systems. It was conceived as an opportunity to present our research aim—how to make lived-experience research more well-known and accessible. In addition to obtaining general support for our aim, we had the opportunity to begin to move into the ideation stage. Together, workshop participants created an inspirational space—the problem or opportunity that motivates people to search for solutions. Small working groups ideated about the potential ways in which lived-experience research could be communicated in lay-friendly ways. This included: (i) making tangible products, (2) talking, telling and explaining and (3) acting, enacting and playing. The two-hour session was facilitated by co-author H.G.

#### Bringing Together Lived-Experience Research—Selecting the Research Papers

Equipped with knowledge from the first two steps, the research team then moved to the selection of the research papers to be translated into user-friendly resources. We initially focused on peer-reviewed papers only, to demonstrate that we are using high-quality research (without assuming that only/all peer-reviewed research is actually high quality). The papers we searched for included research papers (excluding anecdotal, review, conceptual, or opinion pieces), from the peer-reviewed literature (excluding reports, books, etc.) and with lived-experience authorship. A further criterion was that the topic needed to have relevance and clear implications for our end-user group (i.e., not addressing clinicians or researchers). Our team acknowledged the abundance of valuable work that does not fit our established criteria, that might be of potential use to those with lived experience. However, we also wanted to balance this with the need to adhere to fairly strict parameters, as we were mindful of our limited resources. 

Initially, it was assumed that there would be sufficient rich and appropriate papers within the peer-reviewed literature to enable creation of resources that would be most useful to our target group, but we found a lack of papers in the peer-reviewed literature that had obvious and direct implications for individuals with lived experience. It is perhaps unsurprising that many articles focus on implications for clinicians, system designers, policy makers and researchers, given journal readership. Consequently, the requirement that articles be peer-reviewed was revised, as peer review was deemed to be less important than topical relevance to people’s recovery process. This allowed inclusion of material from doctoral dissertations (which have undergone review), reports or book chapters, if similar content could not be found in a peer-reviewed article. In the end, all selected articles had been peer-reviewed, thus we were confident in their quality.

The process of identifying lived-experience research relevant to individuals with lived experience was not without its challenges. We concentrated on articles that highlighted strategies that individuals could consider to help with their recovery journey. The major feature was that they contain practical tips and coping strategies or suggest different ways of thinking, that individuals with lived experience could use in their daily lives (without necessarily having access to particular services or resources). Once the articles were identified, each article was summarised according to a uniform format to identify the main messages in clear, lay language. These summaries were then presented to peer workers and individuals with lived experience via a survey (on SurveyMonkey and via Facebook) to rate each article in terms of usefulness and identify those that resonated in importance and implications for the recovery process. The questions asked of ten selected articles were: select the top three that interest you, article would be useful in day-to-day life/recovery and the potential for the results/main messages to be translated in an engaging, user-friendly manner. Based on the survey findings, six research studies were identified to develop into user-friendly resources (Table 1). The lived-experience authors from each of the six papers were next emailed to obtain the extent of their involvement in the paper, ensure they were happy for the article to be used and to read and comment on the summary we developed.

### 3.3. Phase 3: Ideation: Developing Creative Ways to Bring Lived-Experience Research to Those Impacted by Mental Illness/Distress

Phase 3 of the design thinking process focuses on ideation, facilitating creative opportunities for a diverse range of people to brainstorm and generate a range of ideas that could more effectively disseminate lived-experience research knowledge. We began this process with the workshop outlined in phase 2, which allowed us to identify a preliminary range of creative solutions. This was followed by a day-long design thinking lab, where design students from a local university were recruited as part of their internship program, to assist with the design, prototype and production of resources alongside the research team and peer workers. The composition of the design thinking lab was as follows: five research team members, four design students, six individuals with lived experience, five peer workers and two members of the general public. Author H.G., with experience in facilitating design thinking workshops and in writing about design thinking methods, facilitated the day. Engaging the group in this form of prototype testing encourages intervention solutions that match the contexts of the people and organisations that will be using and implementing the resources [19]. The team also ensured that suggested solutions were informed by the evidence base in knowledge translation [13]. The purpose of ideation is to generate an abundance of strategies for solving problems by targeting multiple factors. The methods we used (e.g., creative matrix) were intended to purposefully encourage both pragmatic strategies and unanticipated, novel, “blue sky” solutions. Subsequent design thinking methods (e.g., importance-difficulty matrix) were used to prioritize solutions with the greatest feasibility and impact.

The design thinking lab began with an introduction to the research project, background and purpose. This was followed by presentation pitches of the topics identified in the review, topic selection and organisation of teams to address each topic. The 90 s pitch for each topic was created to help workshop participants choose what they wanted to focus on. Each research team member had previously prepared a 90 s pitch of approximately 150–180 words on the six topics. The pitch explained the topic, why the area might be useful to people with lived experience, the problem/question and why individuals might want to engage in this particular topic.

Six design teams were formed, focusing on an article of choice. Each design team responded to the ‘How might we…’ question, namely, how might we create accessible, exciting, interesting, engaging resources for people experiencing mental health issues to know and benefit from lived-experience research? Each small design team contained at least one design student and one member of the research team. Design students brought in their relevant transdisciplinary expertise (nursing, marketing and communications, creative design) and outside thinking strategies. The design groups were introduced to the basics of design thinking (including the associated parameters, that is, desirability, feasibility and viability), and considered practical design constraints such as time and money (an upper limit of $500AUS was given per resource) required to produce the resource. The groups began by spending about 30 min reading the summary pages of information on their chosen topic (plus the original resources available if they needed to refer to them) and identifying the preliminary take-home messages from these.

Personas are essential in the human-centred design process to aide designers in honing their designs specifically to the needs and demographics of those they are designing for [17]. The design teams created two personas of an end-user of their designs, based on researcher knowledge and lived experiences. Each design team then divergently brainstormed ideas, without limits, before sorting and converging design possibilities down to those that were most desirable to the persona, feasible to create and viable to disseminate. This was followed by a rapid prototyping session that involved a process of draw–make–test–break–redo. This included feeding back to the larger group at various stages for ideas and feedback. Our aim was to have a full prototype for each of the six topics by the end of the day. Each group then presented their prototype to the group and handed it back to the team with recommendations of what else was needed to carry it forward to implementation.

Following the design lab, the ideas and prototypes were taken up by the design students for further development. The design students devised and produced the resources with regular input and feedback on both content and format from the research team and peer workers. Some of the designs changed significantly as they were further iterated by design students, peer workers and the research team. Table 2 summarises the resources developed for each of the six topic areas.

The final phase in design thinking is to trial the resources, which were introduced and facilitated by peer workers to people in inpatient and community mental health settings. This component of the project is described in detail elsewhere [14]. The use of peer workers was considered both important and consistent with the evidence base, acknowledging that the insights of individuals who have experienced mental health issues are fundamental to recovery frameworks. Peer support workers facilitate egalitarian spaces for non-peer staff and individuals with lived experience to candidly discuss the lived experience of mental illness [25] and can promote recovery at an individual and organisational level [26]. In brief, we used a non-randomised experimental design in two local area mental health services, where five peer workers introduced the resources over an eight-week period of time. Relevance and helpfulness of the resources were assessed before, during and after using standardised measurement tools and in-depth interviews. Although the resources were designed as stand-alone resources, in this first instance, we wanted to trial them with the assistance of a peer worker, who could facilitate the process by introducing individuals with lived experience to the resources, explain them and follow-up regarding use.

The implementation phase provided the research team with vital feedback and insights to iterate the resources even further, prior to any further dissemination and implementation. Iterations covered clarity of language, product format, explanation of where the resources came from and how they link to each other. Additional iteration of the resources is currently being undertaken so they can be scaled to be downloaded and produced to be used by peer provider groups.

## 4. Discussion

The mental health lived-experience research base provides a plethora of collective experiential wisdom to our mental health knowledge. As is the case with a great deal of academic literature, this knowledge is not easily available, accessible or even known to people who are living day-to-day with the impacts of mental distress. Knowledge translation of existing research that is driven or determined by lived experience can play a significant role in assisting people to navigate both their recovery and the systems of care that they may be accessing. Most approaches to sharing knowledge are linear and fail to constantly test to see if they are actually relevant in the ‘real world’ [27]. In the process of connecting to lived-experience research and drawing upon design thinking, we were able to engage in an iterative process to continually refine the resources.

Design thinking is an iterative and solutions-based process that shares many features with an integrated knowledge translation approach [28]. They both involve engaging stakeholders or knowledge end-users as collaborators throughout the project, address needs or priorities identified by end-users, lead to the co-creation of solutions with end-users, can be messy, non-linear and challenging, can lead to unique insights and can improve relevance to end-users.

## 5. Conclusions

Co-design approaches can help researchers and stakeholders to create meaningful, acceptable and novel interventions that target the complex, multilevel factors influencing the communication of evidence-based knowledge. This research adds to the knowledge base on the use of participatory design thinking strategies to enhance knowledge translation to peer workers and others impacted by mental health issues. The resources developed in this co-creation process will deliver (via peer workers) main messages and evidence-based experiential wisdom in areas of personalized medicine, physical health, hope, meaningful activities and recovery.

## Figures and Tables

**Table 1:** The topics.

Topic	Description
1. Concepts of recovery	Recovery means different things to different people. This research examined different ways people thought about recovery and what difference it made to them.
2. What helps recovery?	This research identified the many different strategies that people with lived experience said helped them in their recovery.
3. Hope	Hope is critical for recovery. This research described how people with lived experience are active in finding and maintaining hope.
4. Personal medicine	Personal medicines are non-pharmaceutical strategies that people use to help them get and stay well. This research investigated how people develop and use personal medicine.
5. Physical healthcare	Sometimes people who experience mental health issues have trouble getting proper care for physical health problems. In this research, individuals with lived experience identified strategies they used to get good physical healthcare.
6. Meaningful activity	This research identified the ways in which engaging in activities that are meaningful can help to change how one thinks and feels.

**Table 2:** Resources developed.

Topic Area	Article Reference	Format
1. Concepts of recovery	Factors consumers identify as important to recovery from schizophrenia [1]	Podcast
2. What helps recovery?	Mental health recovery: What helps and what hinders? [20]	Portraits with quotes
3. Personal medicine	The importance of personal medicine: A qualitative study of resilience in people with psychiatric disabilities [21]	Workbook
4. Hope	Igniting and Maintaining Hope: The Voices of People Living with Mental Illness [22]	Gift box
5. Physical healthcare	Mental health consumer experiences and strategies when seeking physical healthcare: A focus group study [23]	Card deck
6. Meaningful activity	Coping with mental health issues: Subjective experiences of self-help and helpful contextual factors at the start of mental health treatment [24]	Magazine

## Data Availability

Not applicable.
